# A Unified Framework for Activity Recognition-Based Behavior Analysis and Action Prediction in Smart Homes

**DOI:** 10.3390/s130202682

**Published:** 2013-02-22

**Authors:** Iram Fatima, Muhammad Fahim, Young-Koo Lee, Sungyoung Lee

**Affiliations:** Department of Computer Engineering, Kyung Hee University, Yongin-Si 446-701, Korea; E-Mails: iram.fatima@oslab.khu.ac.kr (I.F.); fahim@oslab.khu.ac.kr (M.F.); sylee@oslab.khu.ac.kr (S.L.)

**Keywords:** activity recognition, smart homes, behavior analysis, action prediction, lifestyle analysis, u-healthcare

## Abstract

In recent years, activity recognition in smart homes is an active research area due to its applicability in many applications, such as assistive living and healthcare. Besides activity recognition, the information collected from smart homes has great potential for other application domains like lifestyle analysis, security and surveillance, and interaction monitoring. Therefore, discovery of users common behaviors and prediction of future actions from past behaviors become an important step towards allowing an environment to provide personalized service. In this paper, we develop a unified framework for activity recognition-based behavior analysis and action prediction. For this purpose, first we propose kernel fusion method for accurate activity recognition and then identify the significant sequential behaviors of inhabitants from recognized activities of their daily routines. Moreover, behaviors patterns are further utilized to predict the future actions from past activities. To evaluate the proposed framework, we performed experiments on two real datasets. The results show a remarkable improvement of 13.82% in the accuracy on average of recognized activities along with the extraction of significant behavioral patterns and precise activity predictions with 6.76% increase in F-measure. All this collectively help in understanding the users” actions to gain knowledge about their habits and preferences.

## 1. Introduction

A smart home is an intelligent environment that proactively and sensibly perceives the state of residents and the physical environment using sensors. It is one of the best solutions allowing the provision of monitoring and health assistance for persons with special requirements and the elderly to receive services in their own home environments within the limits of their abilities [1]. In the recent years, several smart homes have been developed such as MavHome [2], CASAS smart home [3], Aware Home [4], The Adaptive House [5], House_n [6] and House A [7]. The aim of this smart home technology is to provide ambient assisted living for care delivery, remote monitoring, early detection of problems or emergency cases and promotion of residential safety and quality of life [2–7]. The advancement of sensor technology has proven itself to be robust, cost-effective, easy to install and less intrusive to fulfill the needs and preferences of inhabitants and respond intelligently in seamless and unobtrusive manner. For this purpose, several machine learning-based probabilistic and statistical algorithms have been previously used to identify the performed activities with acceptable accuracy according to their specific intentions [8–11]. With the quest of developing more accurate classification for activity recognition, nowadays, researchers have realized the importance of utilizing the recognized activities for a list of other applications such as behavioral analysis, lifestyle prediction, interaction monitoring, and security and surveillance [12–14]. In order to provide these services, the environment should learn about the routines and habits of inhabitant from the patterns of performed activities. Therefore, activity recognition-based behavior analysis and lifestyle prediction is an important research topic to identify significant behaviors and life threatening complications in the daily routines of inhabitants marked by deviations in normal activity patterns and forthcoming actions (we use the terms activity and action interchangeably throughout the paper).

Usually, inhabitants perform routine actions in a sequential manner characterized by preceding and following activities to identify their influence on each other [15]. For example, taking medicine is very likely followed by eating, and brushing teeth is usually preceded the face washing activity. Therefore, the activity log in terms of performed activities can be effectively analyzed to discover the sequential behavior patterns. The identified patterns provide the significant list of action that mostly occurs together in daily routine to support the health maintenance and functional capability of individuals. For example, in the daily routine of inhabitant Mr. Ben, if the significant sequential behavioral pattern is: (wakeup, exercise, bathing, breakfast, medication), this it reflects that Mr. Ben's activities includes daily exercise before breakfast and he is on constant medication. In this case, the care givers can easily identify the missing exercise and medication routines after analyzing his lifestyle based on frequently performed activities. Furthermore, assuming that human beings perform behaviors based on habits, it could be inferred that patterns describing past and present behaviors will define future actions as well. Thus, learning of user behavior by means of a sequence of actions is highly desirable and is not yet available. The prediction about future actions allows caregivers to take proactive actions for the wellbeing of inhabitants after analyzing their healthy or unhealthy routines. Thus, according to the routine of Mr. Ben, after his exercise activity the most likely activity is of having a breakfast and the framework can remind him to measure his blood pressure and heart rate just before breakfast, if required. However, there is a shortage of formal, systematic and unified behavior modeling and analysis methodologies based on daily life activities. So far, most of the existing applications [8–14] relate an action to the set of sensor values instead of relating the actions among themselves.

Motivated by the lack of a comprehensive approach in smart home-based lifestyle analysis, in this paper we propose a novel and unified framework to analyze user behaviors and predict future actions by using daily life activities. For this purpose, first, we improve the accuracy of activity recognition by adopting a decision fusion mechanism through multiple Support Vector Machine (SVM) kernels [11]. The proposed method transforms the activity recognition problem into higher features space by combining the output of each individual kernel for the final consensus about the activity class label. Our approach is able to recognize activities more efficiently in a reasonable amount of time using a fast Sequential Minimal Optimization (SMO) training method instead of Quadratic Programming (QP). Furthermore, for behavioral analysis, we extract the behavioral pattern from the day to day performed activities in a sequential manner with the help of data mining techniques. We apply the SPAM [16] sequential pattern mining algorithm by modifying it according to the requirements of behavior modeling from the activity log. In our proposed framework, each sequence is a set of activities performed in a temporal order of three days for consistent sequence prediction. Finally, the sequential activity trace is utilized for behavior learning to predict the future actions. A Conditional Random Fields (CRF) algorithm is designed for ongoing activities as labeled sequences and future actions as observations. Therefore, the analysis of the history information transmitted by users” activities helps in discovering the routine behavior patterns and future actions of inhabitants in a home environment. For empirical evaluation, we performed experiments on two real datasets from the CASAS smart home [3]. The results show that our proposed framework first yields a significant improvement in accuracy for the recognized activities as compared to the single kernel function. Then the identification of significant behavioral sequential patterns and precise action prediction enables the observation of the inherent structure present in users” daily activity for analyzing routine behavior and its deviations.

The rest of the paper is organized as follows: Section 2 provides information about some of the existing approaches. Section 3 presents our proposed framework for activity recognition-based behavior analysis and action prediction in smart homes. In Section 4, we illustrate the experimental results followed by comparison and discussion. Finally the conclusion and future work are drawn in Section 5.

## 2. Related Work

In recent years, significant approaches for activity recognition have used audio/video sensors [17], wearable sensors [18,19] and embedded sensors [20,21]. However, several problems are associated with the first two approaches; for instance, audio/video sensors are not practical due to privacy issues, require a large storage space for recording streams, and their accuracy also depends on appropriate location of the sensor while considering the day/night vision problems and the complex environment. Wearable sensors are uncomfortable and inconvenient for users, and their accuracy depends on the body attachment position. Embedded sensors are an acceptable solution for sensing the environment (e.g., smart homes) without disturbing inhabitant privacy and without adding the extra burden of wearing sensors. Therefore, several studies have been conducted to determine effective and accurate activity classification-based behavior analysis methods for smart homes. Rashidi *et al.* [15] tracked the regular activities to monitor functional health and detect changes in an individual's patterns and lifestyle. They described an activity mining and tracking approach based on Markov models and validated their algorithms on data collected in physical smart environments. Similarly, Kasteren *et al.* [22] used a probabilistic model dynamic Bayesian network using a less parametric approach to give better results. They showed how the use of a sensor observation history increased the accuracy in the static model case. Furthermore, the use of the observation history allowed their model to capture more correlations in sensor patterns. Nugent *et al.* [23] analyzed the user's interaction with technology and environment in order to provide useful information relating to lifestyle trends and how the environment can be adapted to improve the user's experience. They proposed homeML, an XML based cross-system standard, to support information exchange between intra- and inter-institutional levels. Their proposed XML-based schema improved the accessibility and analysis of the collected data for meaningful analysis of person's life within smart home environments. Rashidi *et al.* [24] applied data mining techniques to solve the problem of sensor selection for activity recognition along with classifier selection in smart homes. They examined the issue of selecting and placing sensors effectively in order to maximize activity recognition accuracy. Chikhaoui *et al.* [25] applied sequential pattern mining for person identification in a multiuser environment. Their proposed approach is utilized for audiovisual and image files collected from heterogeneous sensors in smart homes.

Fusion techniques play an important role to achieve high accuracy as compared to single classifiers and successfully produced more accurate results in different application domains such as image processing [26], and gene functional classification [27]. In the context of activity recognition, Xin *et al.* [28] addressed the fusion process of contextual information derived from the sensor data. They analyzed the Dempster-Shafer theory and merged with a weighted sum to recognize the activities of daily living. Rongwu *et al.* [29] proposed classifier fusion as a learning paradigm where many classifiers are jointly used to solve the prediction problem. They used seven wearable sensors including five accelerometers and two hydrophones. Their used classifiers are Linear Discriminant Classifier (LDC), Quadratic Discriminant Classifier, k-Nearest Neighbor (k-NN) and Classification and Regression Trees (CART).

So far, most of the applications where a learning process is involved have treated it as an action to map the overall situation instead of relating the actions among themselves. They process independent pieces of information instead of complete and comprehensive representation of user behavior. However, some of the research groups started to create methods to relate user actions. Fernández *et al.* [30] applied the workflow mining technique to infer human behaviors. Their approach involved an expert user who can identify the changes in behavior of dementia patients. They validated their approach on synthetic data to identify the deviation from normal behavior. Aztiria *et al.* [31] focused on automatic discovery of user behavior as a sequence of actions. Their developed approach is based on discovery of frequent sets, identification of topology and temporal relations of performed activities with other constraints. Doctor *et al.* [32] focused on developing an application based on set of fuzzy rules to represent the users” patterns. They recorded changes caused by users in the smart environment and generated the membership functions that mapped the data into fuzzy rules. A survey of all these works can be found in [33,34]. The focus of all the above mentioned research is to discover the behavior patterns; however a step towards predicting the future actions from a set of performed activities is still need to be explored for better analysis of human lifestyle and intended services.

The methodologies commonly observed in the literature for activity recognition and behavior patterns discovery in smart homes are limited to a number of algorithms in order to select the one which gives relatively better results for a particular domain. Our objective is to overcome the limitation of existing methods by introducing a unified framework for behavior analysis of inhabitants that ranges from activity recognition to action prediction in order to support the smart home inhabitants in performing their daily tasks and providing personalized services adapted to their needs.

## 3. The Proposed Framework

**Figure 1.**
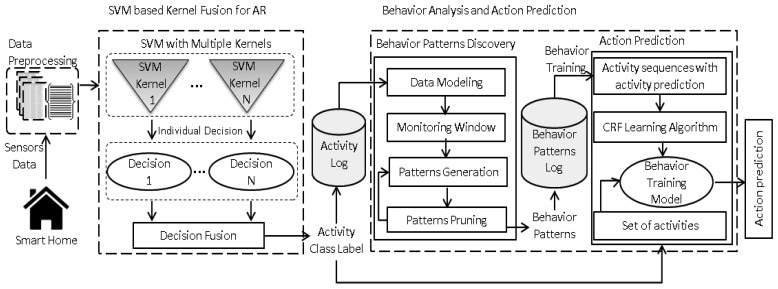
The architecture of the proposed framework.

In the proposed approach, an activity is defined as set of active sensors at a particular time that perform a certain task in a smart home environment. It can be recognized over the collected sensory data and annotated either at the micro level (*i.e.*, book reading) or macro level (*i.e.*, leisure) from the daily life of inhabitants. The proposed framework consists of three major modules, as shown in Figure 1: (1) Data preprocessing: to represent the sensory data as an observation vector for kernel functions. (2) SVM based kernel fusion for activity recognition: to recognize the daily life activities using decision fusion of four individual SVM kernel functions, where each kernel is designed to learn the performed activities in parallel. (3) Behavioral analysis and action prediction: to identify the sequential behavior patterns and then predict the future actions by utilizing the significant behavior of inhabitants” daily life. The details of each module are described in the following sections.

### 3.1. Data Preprocessing

Data preprocessing is an important step towards accurate training in machine learning techniques [35]. Data collected from ubiquitous sensors based on subject interactions are stored in sensor logs and annotation files with attribute start times, end times, sensor ids, sensor values and activity labels. In order to recognize the performed activities, a recorded dataset is preprocessed into the form {(*x*_1_, *y*_1_), …, (*x_n_*, *y_n_*)}. The term “*x_i_*” represents the vectors whose components are the values of embedded sensors such as stove-sensor, refrigerator-sensor, and door-sensor. The values of “*y*” are drawn from a discrete set of classes {1, …, *K*} such as a “Leave Home”, “Read”, and “Sleep”. In addition, excessive information such as multiple header lines is also removed from the sensor logs and annotation files.

### 3.2. SVM based Kernel Fusion for Activity Recognition

SVM is a statistical learning method to classify activities through determination of a set of support vectors and minimization of the average error rate. It can provide a good generalization performance due to its rich theoretical basis and by transferring the problem to a high dimensional feature space [11]. Activity recognition is a multi-class problem so we adopt a “one-versus-one” approach to recognize the performed activities. Given a training set of sensor values and activity pairs (*i.e.*, (*x_i_*, *y_i_*)), we train our model through Sequential Minimal Optimization (SMO) for efficient performance. SMO avoids time consuming matrix calculation by dividing the optimization problem into the smallest possible portions and consequently resolves the memory issues for large training data [11]. The following optimization model is applied by using the Lagrangrian multiplier techniques and Kernel functions:

(1) $$\text{Maximize}\;(w.r.t\alpha )\sum_{i=1}^{n}\alpha_{i}-\frac{1}{2}\sum_{i=0}^{n}\sum_{j=1}^{n}\alpha_{i}y_{i}\alpha_{j}y_{j}K(x_{i},x_{j})$$

(2) $$\text{Subject to}:\sum_{i=1}^{n}\alpha_{i}y_{i}=0,0\leq \alpha_{i}\leq C$$

where *K* is the kernel function that satisfies *K*(*x_i_*, *y_i_*) = Φ*^T^*(*x_i_*)Φ(*x_j_*). It is a function that transforms the input data into a high-dimensional space where the separation could be linear [36]. SVM can provide high performance, but usually far from the expected level of accuracy, due to its approximation algorithm and high data complexity. In order to achieve better accuracy in the classification, we train following multiple kernels [37,38] and fuse the individual results at decision level:

(3) $$K(x_{i},x_{j})=x_{i}^{T}x_{j}$$

(4) $$K(x_{i},x_{j})={\left(x_{i}^{T}x_{j}+1\right)}^{p}$$

(5) $$K(x_{i},x_{j})=\exp\left(\frac{-{\left\|x_{i}-x_{j}\right\|}^{2}}{\left(2\sigma^{2}\right)}\right)$$

(6) $$K(x_{i},x_{j})=\text{tanh}\left(kx_{i}^{T}x_{j}-\delta \right)$$

Equations (3–6) show the linear, polynomial, Gaussian (RBF) and multi-layer perceptron (MLP) kernel functions, respectively. In activity recognition, an ideal kernel function assigns a higher similarity score to sensor observations that belong to the same activity class. However, in real life scenarios, a kernel function behaves differently on the performed activities due to the complex situation of different active sensor events for an individual activity. As a result, a single kernel function is inadequate to perform well for all annotated activities. We trained different kernel functions and fuse the individual results on decision level. Before assigning the final class label, we get the confidence with the help of max rule as below:

(7) $$\text{Activity Label}=\text{argmax}\;\left(\sum_{i=1}^{4}\text{SVM}(K_{i})\right)$$

The recognized activities are stored in an activity log repository and can be effectively utilized by caregivers and service providers to assist the inhabitants adequately after analyzing their lifestyles. Therefore, routine patterns discovery and future actions” prediction are the main sources to acquire knowledge about the inhabitants” lifestyles.

### 3.3. Behavior Analysis and Action Prediction

This module has further two sub-modules: (a) behavior patterns discovery: to discover the behavior patterns by applying the sequential pattern mining algorithm on the activity log data collected from activity recognition module and (b) action prediction: to identify the next actions in a series of consecutive actions. The detail of each sub-module is given in the subsequent sections.

#### 3.3.1. Behavior Patterns Discovery

Representing the inhabitants' actions by means of ordered sequence of activities facilitates our understanding of the significant behavior patterns in daily lifestyles. Therefore, the objective of this module is to identify the set of actions that frequently occur together. One intuitive way for behavior pattern generation is to apply a sequential pattern mining technique. For this purpose, we are given a repository of activity log “*Al*” where activities are stored in sequential order with respect to activity time. Let *D* = {*a_1_*, *a_2_*, …, *a_m_*} is a set of *m* activities performed in a particular day in a temporal manner *T*. Let each sequence in the “*Al*” be *S* = {*D_1_*, *D_2_*, …, *D_n_*}, where *D_i_* is a set of performed sequences of activities on different days. For instance a set of sequential activities is defined as an individual who comes to the bedroom to sleep is likely to read or watch TV before the sleep activity. The sample activity log is shown in Table 1. In our proposed data modeling, the monitoring window is a list of activities performed in three days ordered by activity time.

Here, the problem is to discover all sequential patterns with a specified minimum support, where the support of a pattern is the number of data-sequences that contain the pattern as shown in Equation (8). Therefore, a sequence pattern is a non-empty set of “*Al*” and a day *D_i_* is said to contain pattern *P* if *P*⊆*D_i_*:

(8) $$\text{Supp}(P)=\frac{\text{the number of instances containing P in Al}}{\text{the number of instances in Al}}$$

The pseudocode for the frequent sequential behavior patterns is shown in Algorithm 1 for activity log *Al* and support threshold ∝. Here, *S_k_* is the candidate set for level *k*, genrated by *fGenCanSet*(*Al*) method and *fGenActivitySequence* (*S_k_*) method is assumed to generate the candidate sets *CS* from the large activities of the preceding level. The downward closure *count* (CS) accesses a field of the data structure that represents candidate set *CS*, which is initially assumed to be zero. Therefore, all the activities in an element of a sequential pattern necessarily present in a single day activities for the data-sequence to support the pattern. A pattern is regarded as persistent if it has the highest support. This demonstrates the most significant behavior of inhabitant due to its high continued or repeated ratio as compared to other identified patterns under same support threshold.


**Algorithm 1:** Frequent Sequential Behavior Patterns
**Input:***Al*: Activity log∝: Support threshold**Output:***Bp*: Behavior patterns**Begin**1*S_1_* = *fGenCanSet*(*Al*)2*k* = 23While (S_k-1_! = *Null*)4 *CS* = *fGenActivitySequence*(*S_k_*)5 for *j* = 1:*length* (*CS*)6  if (*Supp*. (*CS* (*j*)) > ∝)7   *Count* (*CS* (*j*)) = *Count* (*CS* (*j*)) + 18   *S_k_* = *CS*(*j*)9   *k* = *k* + *1*10  end11 end12 *Bp* = *Union*(*S_k_*)13end**End**


The analysis of frequent user behaviors *Bp* reveals the significant habits of inhabitants from their daily routines and provides the basis for behavior learning to predict their future actions.

#### 3.3.2. Action Prediction

**Figure 2.**
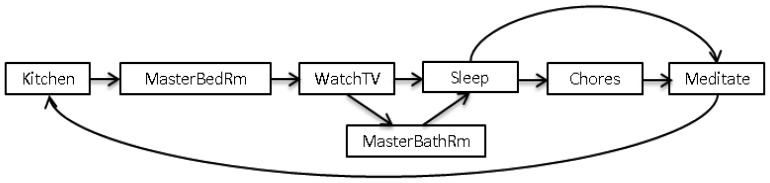
Set of sequences with activity relationships.

The objective of this module is to predict the next action from the set of activities that occur together. For the learning process of action prediction, required data is extracted from the behavior pattern log. Let”s consider activities shown in Table 2 are occurring together in different sets of actions and the same set of activities with their relationships among them is presented in Figure 2. It is obvious that the occurrence of each activity depends on the set of previous actions. For example, “Meditate” comes after “Sleep” or “Chores”, whereas, “Sleep” comes after “Watch TV” or “Master Bathroom” and there could be the repetitive actions in the same sequence. Therefore, a decision about the next activity depends on the transition of previous actions. For instance, “Kitchen” activity follows by “Meditate” or “Sleep” represents breakfast while “Kitchen” activity after “Enter Home” represents dinner. So it is clear that a set of previous actions provide remarkable evidence to identify the meaningful behavior in terms of forthcoming action. In our proposed approach, sequences of 8 to 10 activities are considered to predict the next action.

**Figure 3.**
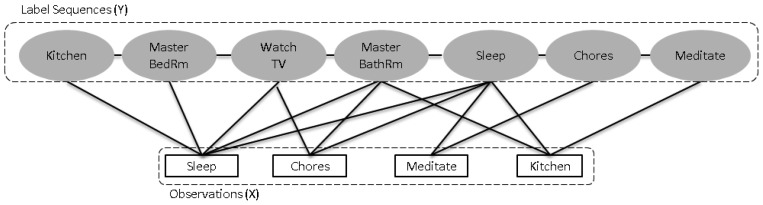
The design of CRF for activity sequences.

Once the sequences of activities are selected, we utilize them for the learning process of action prediction. Prediction in smart environments is a challenging task and a variety of machine learning algorithms are available for effective learning for a particular domain [11–18]. In our proposed framework, we choose CRF as a learning classifier for predicting the future actions. It is a discriminative and generative probabilistic model for labeling the sequences under the conditional probability *p*(*y*|*x*). It is modeled as undirected acyclic graph that allows arbitrary, non-independent relationships among the observation sequences. A CRF flexibly capture the relation between a pair of observations and label sequences that do not explicitly model the marginal probability of observations. It uses a potential function instead of a joint probability. Suppose there are finite label sequences *Y* = (*y1*,*y2*,…,*yT*−*1*,*yT*) and observations *X* = (*x1*,*x2*,…,*xT*). In Figure 3 a design of CRF is shown for the activity sequences presented in Table 2.

In the CRF model, the conditional probabilities of next action with respect to previous activity observations are calculated as follows:

(9) $$p(y_{1:T}|x_{1:T})=\frac{1}{Z(x_{1:T},w)}\text{exp}\left\{\sum_{j=1}^{N_{f}}w_{j}F_{j}(x_{1:T},y_{1:T})\right\}$$

In Equation (9), *Z* denotes normalized factor and *F_j_*(*x*_1:T_,*y*_1:T_) is a feature function that is computed as:

(10) $$F_{j}(x_{1:T}|y_{1:T})=\sum_{t=1}^{T}f_{j}(y_{t-1},y_{t},x_{1:T},t)$$

In Equation (10), the feature function depends on known observations *x*_1:_*_T_* and is determined by any combination of input values instead of considering all arguments. To make the inference in the model, we compute the most likely activity sequence as follows:

(11) $$y_{1:T}^{*}={\text{argmax}}_{{\text{y}}_{1:\text{T}}^{\prime }}p({\text{y}}_{1:\text{T}}^{\prime }|x_{1:T},w)$$

Hence, the learning capability of CRF in terms of sequences of actions is able to capture long-range transition among activities collected from behavior patterns log for future action prediction.

## 4. Evaluation and Results

In this section, we present the results to evaluate and validate our proposed framework to measure the accuracy level of recognized activities, usefulness of behavior patterns and reliability of action prediction.

### 4.1. Datasets Description

The experiments are performed on the *Milan2009* and *Aruba* datasets collected in the CASAS smart home, a project of Washington State University, with full-time residents [3]. In the case of *Milan2009*, 31 motion sensors, one door sensor, and two temperature sensors were deployed at various locations and 15 activities were performed for 62 days. For *Aruba*, 31 motion sensors, three door sensors, five temperature sensors, and three light sensors were deployed and 11 activities were performed for 220 days. The details description of the datasets and annotation method can be found in [3].

In Table 3, the characteristics of the *Milan2009*, and *Aruba* dataset are shown. The “Num.” column shows activities count, “Time” column shows the time in seconds and “Sensor” column shows generated sensor events.

### 4.2. Performance Measures

In order to evaluate our proposed framework, four standard metrics of precision, recall, F-measure and accuracy are used as performance measures. They are calculated using the values of the confusion matrix [39] and computed as:

(12) $$\text{Precision}=\frac{1}{Q}\sum_{i=1}^{Q}\frac{{\text{TP}}_{i}}{{\text{Nl}}_{i}}$$

(13) $$\text{Recall}=\frac{1}{Q}\sum_{i=1}^{Q}\frac{{\text{TP}}_{i}}{{\text{NG}}_{i}}$$

(14) $$\text{F}-\text{Measure}=\frac{2\cdot \text{precision}\cdot \text{recall}}{\text{precision}+\text{recall}}$$

(15) $$\text{Accuracy}=\frac{\sum_{i=1}^{Q}{\text{TP}}_{i}}{\text{Total}}$$

where Q is the number of performed activities, TP is the number of true positives, NI is the total number of inferred labels and NG is the total number of ground truth labels.

### 4.3. Experiments and Discussion

**Figure 4.**
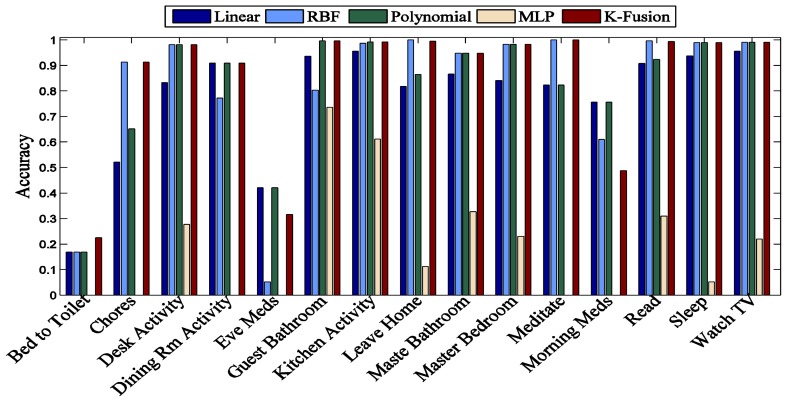
Individual class accuracy of different kernel functions for *Milan2009.*

In this section, first we evaluate the kernel fusion method for recognizing the daily life activities. We split the dataset using the “leave one day out” approach; therefore, the sensor readings of one day are used for testing and the remaining days for training. As can be seen from Figure 4, our proposed model achieves a significant improvement for *Milan2009* in all recognized activities except “Eve Meds” and “Morning Meds” in comparison to individual kernel functions.

It is obvious from the results that different kernel functions show performance variations for the recognition of the same activities. The overall performance of “MLP” is low as compared to other individual kernel functions and in some cases such as “Bed to Toilet”, “Chores”, “Dining Rm Activity”, “Eve Meds”, “Meditate” and “Morning Meds”, “MLP” fails to identify even a single instance of the corresponding activity class label. The performance of the “Linear” kernel function is better than “MLP” for all recognized activities, however its overall performance is not significant even for the identification of single activity class label. “RBF” is good for the recognition of “Chores”, “Leave Home”, “Meditate” and “Read” activities, while “Polynomial” is better than “RBF” in recognition of “Dining Rm Activity”, “Guest Bathroom” and “Morning Meds” activities. Therefore, this comparison represents that the proposed “kernel fusion” outperforms all individual kernel methods for overall accuracy and shows similar or high class level accuracy.

**Figure 5.**
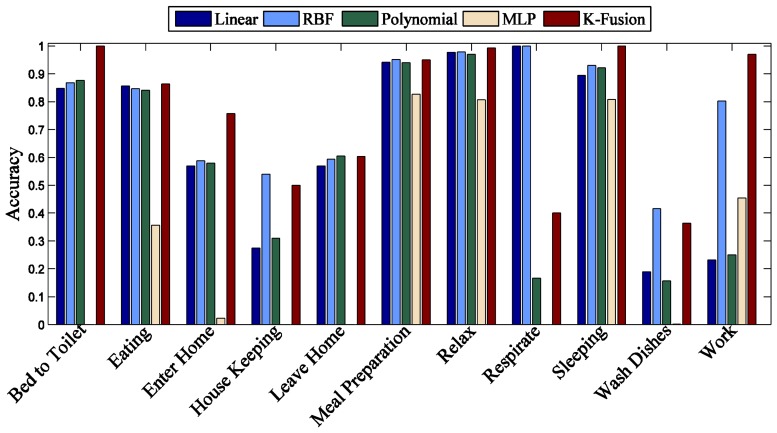
Individual class accuracy of different kernel functions for *Aruba.*

Similarly, for *Aruba* the kernel functions show variation in their performance for the recognition of different activities, as shown in Figure 5. However, a remarkable improvement is achieved by our proposed “kernel fusion” method for the recognition of all activities except “House Keeping” and “Wash Dishes” compared to “RBF”. The “Linear” outperforms others kernel function in overall accuracy however “RBF” and “Polynomial” are better than “Linear” in recognition of some activities such as “Bed to Toilet”, “Enter Home”, “Leave Home” and “Sleeping”. The performance of “RBF” shows its significance as compared to others kernel functions for the recognition of “House Keeping”, “Wash Dishes” and “Work” activities. The overall low accuracy of “MLP” represents its inability to identify a list of activities. It is able to recognize only “Eating”, “Enter Home”, “Meal Preparation”, “Relax”, “Sleeping” and “Work” activities. The overall accuracy of “kernel fusion”, 94.11% (*Milan*) and 92.70% (*Aruba*), is significantly better than that of the individual kernel functions for both the datasets, as shown in Table 4.

**Figure 6.**
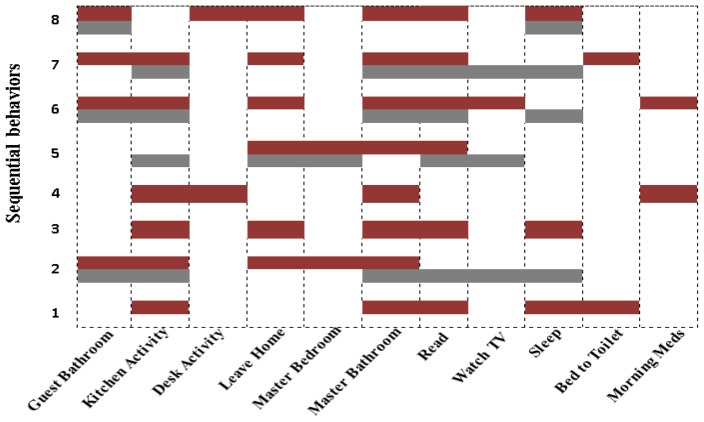
Sequential behavioral patterns for *Milan2009*.

**Figure 7.**
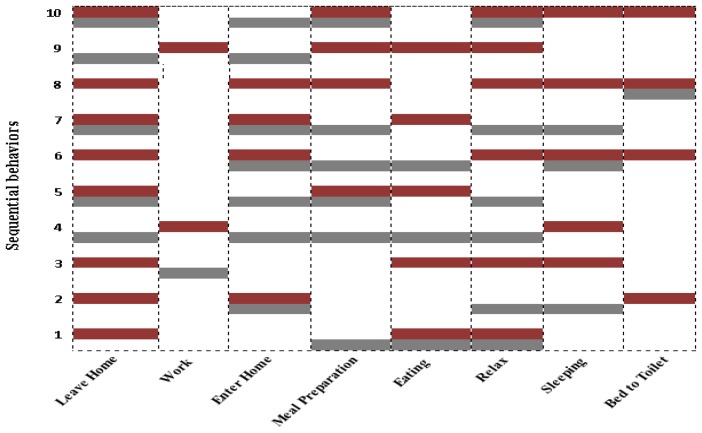
Sequential behavioral patterns for *Aruba.*

The above results and statistics clearly show that dataset characteristics highly affect the “kernel functions” individual class level accuracy and thus their overall performances. In the case of *Milan2009* “Polynomial” outperforms other kernel functions and for *Aruba* the performance of “Linear” is better than others. However, our proposed “kernel fusion” outperforms all kernel function and validates that fusing the individual decisions strengthens the confidence to assign the final activity class label. Once activities are recognized, they can provide the basis to analyze the behavior of individuals from their daily routines. For clear understanding of user behavior, we represent 8 to 10 of the most significant sequential behaviors for *Milan2009* and *Aruba* in Figures 6 and 7, respectively. Here, red and gray bars show sequential patterns in monitoring windows of three days with user specified support. In both the datasets the annotation frequency of activities varies significantly as shown in “Num.” column of Table 3. To discover the sequential patterns we set the support to 0.8% in order the get the most significant behaviors of daily life after patterns pruning. As a result the activities that are annotated with low frequency cannot be identified in the sequential behavior patterns. For instance, “Chores”, “Dining Rm Activity”, “Eve Meds” and “Meditate” have very few occurrences in the *Milan2009* data. Similarly, the occurrences of “House Keeping”, “Respirate” and “Wash Dishes” in *Aruba* are much fewer compared to the other annotated activities.

It is obvious from Figure 6 that in sequential behavior the existence of “Kitchen Activity”, “Master Bathroom” and “Read” are more noticeable due their high annotation ratio in the dataset. The performed experiments on the *Milan2009* activity log represent that the most prominent user behavior such as “Read” activity symbolizes that the user habit of reading before sleep is significant as compared to “Watching TV”. Similarly, “Kitchen Activity” shows its sequence prior to “Desk Activity” and “Leave Home” that represents the users” eating behavior. Furthermore, “Bed to Toilet” and “Master Bathroom” activities show bathroom usage habits before and during “Sleep”.

In Figure 7, a list of ten most significant sequential behaviors is shown for *Aruba*. The most obvious activities are “Enter Home”, “Meal Preparation”, “Relax” and “Leave Home”. The behavior analysis of the *Aruba* activity log shows the most substantial activities in the user routine such as “Meal preparation” illustrate the user's habit of cooking after coming home. The “Relax” activity represents his behavior of relaxing before sleep and “Bed to Toilet” characterizes user's habit to go to the toilet during sleep. Although, the “Work” activity is not noticeable in most of the activity patterns but “Enter Home” activity after “Work” signifies that user go out of home for “Work” activity most of the times. The above frequent sequential patterns can be effectively utilized to analyze the lifestyle of inhabitants in terms of significant routine discovery. Furthermore, these routine behaviors facilitate the personalized service providers (*i.e.*, caregivers) to estimate the forthcoming action of inhabitants in order to take proactive actions for their better lifestyle.

**Figure 8.**
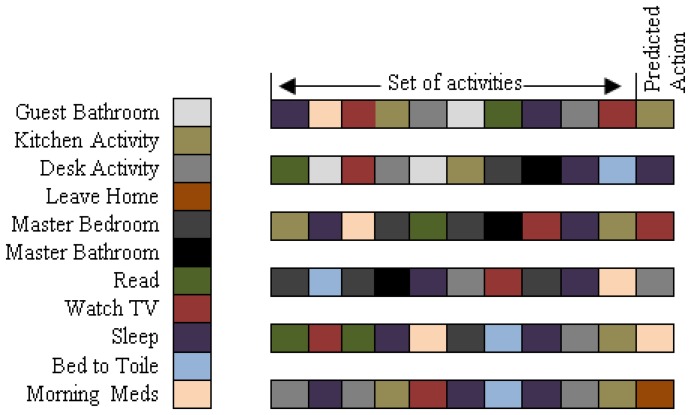
Behavioral predictions for *Milan2009.*

**Figure 9.**
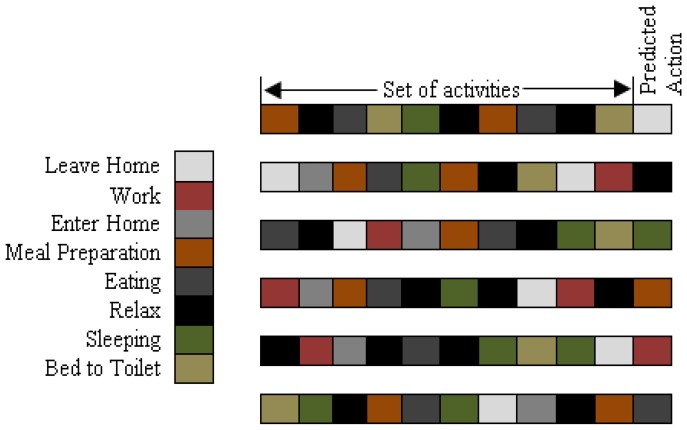
Behavioral predictions for *Aruba.*

To evaluate the activity prediction method, we use the classification of future actions from the past sequential behaviors. The goal of the experiments is to determine how well our method performs in predicting the future actions of inhabitants. In our proposed approach, the sequences of 8 to 10 activities are consider to predict the next action. The representative activity sequences with predicted activity for *Milan2009* and *Aruba* are shown in Figures 8 and 9, respectively.

Here, each activity is symbolized by a specific color and “set of activities” represents the routine behaviors in different sequences and “predicted action” represents the outcome for the particular behavior. For example, in the daily routine of the inhabitant in *Milan2009*, activities prior to “Sleep” are “Master Bedroom”, “Bed to Toilet”, “Watch TV”, and “Read”, and subsequent activities to “Sleep” are “Kitchen”, “Morning Meds” and “Desk Activity”. This represents that prediction of “Sleep” as a forthcoming action depends on the order of priory performed activities as shown in Figure 8. Similarly for *Aruba*, “Sleep” activity is in-between the “Eating”, “Relax”, “Bed to Toilet”, and “Meal Preparation”, so the prediction for “Sleep” as a future action depends particularly on the order in which these activities are performed in the daily routine of the inhabitant, as shown in Figure 9.

We compare our proposed method with the results of HMM [8] which is a well known generative probabilistic graph model for labeling sequences. The quality of a predicted activity is determined based on how closely the predicted activity resembles inhabitant's real future action. We computed the precision, recall, and F-measure, as shown in Table 5. For both the datasets, the CRF performs better in comparison to HMM, the increase of 6.61% and 6.76% in F-measure is achieved for *Milan2009* and *Aruba* respectively.

## 5. Conclusions and Future Work

Personalized service providers need to know the common behaviors and preferences of the inhabitants in leveraging the use of smart home technology for different application domains. In this paper, we proposed a unified framework for activity recognition-based behavior analysis and action prediction. This informs the service provider about inhabitants” significant behavior in order to perform meaningful interventions. In the proposed framework, first, we introduced the kernel fusion method to overcome the learning effects of different kernel functions for recognition of individual activities. Furthermore, the recognized activity log is utilized for behavioral pattern discovery with the help of frequent sequential mining technique on a set of activities that are performed in a temporal sequence of three days. Finally, we investigated CRF for the actions that occur together in order to predict the next activity from a current situation. Our study found that identification of behavior patterns and prediction of forthcoming action with high precision signifies the possibility of helping people by analyzing the long-term data of one's behavior to fulfill his needs in the current circumstances and in future.

To investigate further, we intend to define our own activity domain specific kernel function to refine the accuracy rate and incorporate the extraction of interleave and parallel activities to understand the user behavior in more depth. Moreover, we aim to design the system for predicting a future situation based on a set of forthcoming actions instead of a single activity.

## Figures and Tables

**Table 1.** Representative repository of an activity log.

**Sequence ID**	**Days**	**Activities**
S1	1	Read, Sleep
	2	Kitchen, Master Bedroom, Read
	3	Kitchen, Master Bedroom, Watch TV
S2	4	Read, Sleep, Chores
	5	Master Bedroom, Read, Sleep
	6	Kitchen, Master Bedroom, Watch TV, Master Bathroom

**Table 2.** Representative sequences from behavioral patterns.

**Sequence**	**Prev. Activity 3**	**Prev. Activity 2**	**Prev. Activity 1**	**Next Activity**
1	Kitchen	MasterBedroom	WatchTV	Sleep
2	—	WatchTV	MasterBathroom	Sleep
3	WatchTV	MasterBathroom	Sleep	Chores
4	—	Sleep	Chores	Meditate
5	MasterBathroom	Sleep	Meditate	Kitchen

**Table 3.** Characteristics of the annotated activities of CASAS smart home datasets.

**Milan2009**							
Activities	Num.	Time	Sensor	Activities	Num.	Time	Sensor
Idle	-	911.233	5760	Evening Medicines	19	10.56	250
Bed to Toilet	89	379.37	1255	Guest Bathroom	330	952.31	10601
Sleeping	96	37,217.9	22172	Kitchen Activity	554	7,526.81	128942
Leave Home	214	4,229.47	4946	Master Bathroom	306	1,946.33	15071
Watch TV	114	5,919.72	23688	Master Bedroom	117	2,168.97	27337
Chores	23	684.82	7587	Meditate	17	109.94	1315
Desk Activity	54	743.74	7628	Morning Medicines	41	45.97	1023
Dining Rm Act	22	330.37	4295	Read	314	10,942.75	50281
**Aruba**							

Idle	-	59,495.15	903669	Enter Home	431	48.84457	2041
Meal Preparation	1,606	12,588.53	299300	Housekeeping	33	670.6926	11010
Bed to Toilet	157	428.833	1483	Leave Home	431	45.75227	1954
Relax	2,919	97,813.58	387,851	Respirate	6	51.38585	571
Sleeping	401	139,659.9	63,792	Wash Dishes	65	465.5383	10682
Eating	257	2,610.955	19,568	Work	171	2,920.759	17637

**Table 4.** Overall kernel functions accuracy.

**Kernels**	**Milan2009**	**Aruba**
Linear Kernel	86.86%	89.60%
RBF Kernel	91.34%	87.85%
Polynomial Kernel	91.90%	88.45%
MLP Kernel	37.88%	62.79%
Kernel Fusion	94.11%	92.70%

**Table 5.** Accuracy performance for action prediction.

**Dataset**	**Model**	**Precision**	**Recall**	**F-measure**
Milan2009	HMM	0.7796	0.7363	0.7574
	CRF	0.8478	0.8006	0.8235
Aruba	HMM	0.7261	0.7356	0.7308
	CRF	0.7971	0.7996	0.7984
